# Crowned dens syndrome combined with cervical disc herniation: A case report and literature review

**DOI:** 10.1097/MD.0000000000045039

**Published:** 2025-10-31

**Authors:** Lan Cheng, Yu Chen, Lianjie Li

**Affiliations:** a Department of Cardiovascular Medicine, Ningbo No. 2 Hospital, Ningbo, Zhejiang, China; b Department of Neurosurgery, Xiangshan Red Cross Taiwan Compatriot Hospital Medical and Health Group, Ningbo, Zhejiang, China; c Department of Neurosurgery, Ningbo No. 2 Hospital, Ningbo, Zhejiang, China.

**Keywords:** case report, cervical disc herniation, Crowned dens syndrome, neck pain, odontoid syndrome

## Abstract

**Rationale:**

Crowned dens syndrome (CDS) is an unusual and frequently underdiagnosed cause of intense neck pain in older people due to the lack of awareness. The clinical presentation often mimics intracranial infections, which may result in unnecessary invasive procedures and the inappropriate use of antibiotics. The patient had a long-standing history of chronic neck pain, and the acute exacerbation was initially misdiagnosed as cervical disc herniation with suspected intracranial infection before the diagnosis of CDS was established.

**Patient concerns:**

We report the first case of an 80-year-old female patient who presented with severe suboccipital neck pain for 10 days, accompanied by restriction of rotational movement of the neck and low-grade fever.

**Diagnoses and interventions:**

Differentiating CDS from other potential causes of neck pain, such as degenerative cervical spondylosis, fracture, or metastatic spinal tumors, is challenging given the patient’s history and clinical presentation. Initially, the patient was diagnosed with cervical spondylosis. However, treatment with pain-relief patches, acupuncture, and physiotherapy did not alleviate these symptoms. Diagnosis of intracranial infection based on laboratory tests and clinical symptoms cannot be ruled out. However, the antibiotics had no effect. Plain cervical computed tomography revealed typical calcification around the odontoid process. Increased levels of inflammatory markers can contribute to the final diagnosis of CDS. Radiography of the knee joints revealed calcium deposits in the cartilage and ligament tissues, with no apparent alteration, deformity, or destruction of the joints. This finding supported the diagnosis. After combined administration of celecoxib, colchicine, and a short course of corticosteroids, the patient’s fever subsided rapidly, and both neck pain and restricted cervical mobility improved significantly. Inflammatory markers, including C-reactive protein and erythrocyte sedimentation rate, decreased rapidly and were within normal limits within 7 days.

**Outcomes:**

There was no indication of recurrence, and C-reactive protein and erythrocyte sedimentation rate levels returned to normal at the 2-week follow-up.

**Lessons:**

CDS should be considered in cases of acute pain in the neck or occipital regions. Moreover, prompt computed tomography of the cervical vertebrae may guide early diagnosis and avoid unnecessary invasive procedures or administration of antibiotics.

## 1. Introduction

Crowned dens syndrome (CDS), initially described by Bouvet et al in 1985,^[1]^ is an uncommon cause of neck pain characterized by limited movement, which occurs in 2% of the patients with acute neck pain. The usual clinical signs of CDS include acute posterior neck pain, stiffness, limited movement, fever, and elevated erythrocyte sedimentation rate (ESR) and/or C-reactive protein (CRP) levels. Neurological symptoms are rare. Other signs may also be observed, including radiating pain toward the upper limbs, headache, and, in some cases, numbness of the limbs or urinary disorders caused by spinal cord compression.^[2]^ Imaging reveals dense, irregular calcium deposits of varying sizes surrounding the odontoid process, predominately on the posterior aspect.^[3]^ Calcium crystal deposits cause irritation, leading to acute pain and restricted neck movement. Cervical disc herniation (CDH) involves displacement of intervertebral disc material that compresses nerve roots or the spinal cord, resulting in symptoms such as neck pain, radiculopathy, and sensory or motor deficits.^[4]^ Radiography typically reveals a posterior protrusion of the disc material, often compressing the adjacent neural structures. Magnetic resonance imaging (MRI) is the reference standard for diagnosis^[5]^ We report a case of CDS combined with CDH and highlight the importance of CDS in older patients with chronic neck pain.

## 2. Case presentation

This study was approved by the Ethics Committee of Ningbo No. 2 Hospital. Written informed consent was obtained from the patient and his family members for the publication of this case report and the accompanying images. This case report was exempt from IRB review. An 80-year-old woman presented with a 10-day history of severe suboccipital neck pain radiating to the right shoulder and back. The pain was severe, persistent, intolerable, and worsened after the neck rotation. The patient reported morning stiffness but denied numbness in the arms, reduced vision, or slurred speech. No clinical signs suggestive of abnormalities in the thoracic or abdominal organs were observed. The patient attributed her symptoms to general neck and shoulder strain and received acupuncture and topical Huoxue plaster treatment in her local hospital. However, the patient’s symptoms did not resolve and she was admitted to our hospital for further diagnosis and treatment.

The medical history included lumbar disc herniation and degenerative cervical spondylosis. Acupuncture and massage are frequently used to treat lumbar intervertebral disc herniations. The patient denied smoking, alcohol consumption, or the use of other psychoactive substances, and had no history of trauma or pseudogout. Her initial vital signs were within normal limits, except for a low fever (37.6 °C). The superficial lymph nodes were not swollen on palpation, and there were no specific rashes. The heart, lungs, and abdomen showed no apparent abnormalities. The patient was conscious and cooperated with the neurological examination. No signs of meningeal irritation or focal neurological deficits were observed. However, the cervical range of motion (rotation < 30°) was limited and painful. Laboratory results were as follows: white blood cell count, 11,400 per μL; hemoglobin 9.8 g/dL; platelets, 25.6 × 10^4^ per μL; CRP, 56 mg/L (normal range: ≤6); and ESR,59 mm/h (normal range: ≤20). The laboratory test results for thyroid function were within normal limits. Screening for autoantibodies, including antinuclear antibodies, anticyclic citrullinated peptide antibodies, and proteinase 3 antineutrophil cytoplasmic antibodies yielded negative results. Rheumatoid factor, urinalysis, and kidney function results were within normal limits.

**Figure 1.**
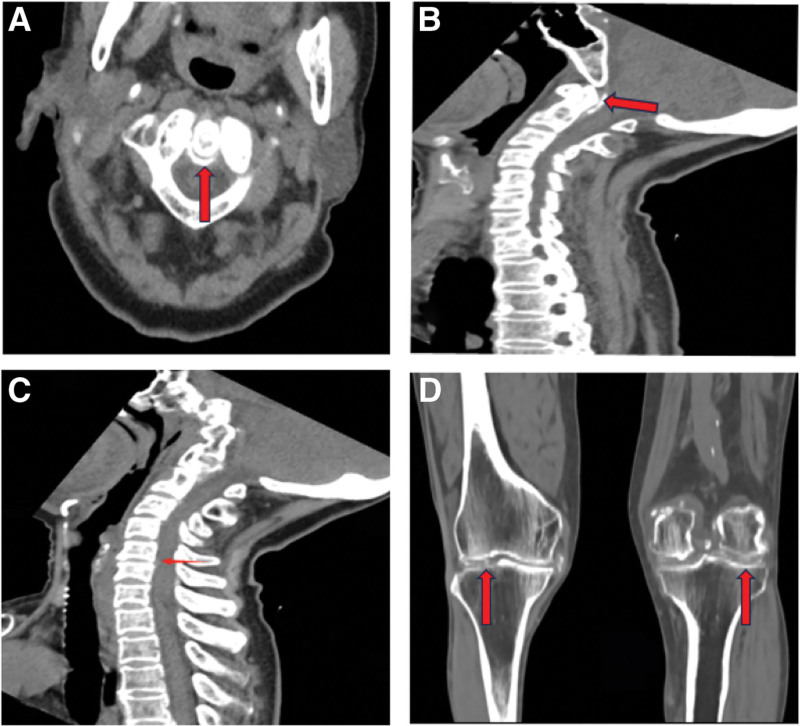
Plain CT-scan of the cervical vertebrate and X-rays of the keen joint. (A) Cross-sectional imaging of the atlantoaxial joint, ring high signal indicated by the arrow; (B) CT sagittal section showed linear calcification; (C) CT sagittal section showed slight posterior bulging of the intervertebral discs of C4/5 and C5/6; (D) X-ray’s of her knee joint.

These results mentioned above indicated that infection could not be ruled out. Therefore, empirical anti-infective therapy with ceftriaxone (2 g intravenously, once daily) was initiated and blood cultures were collected. Unexpectedly, fever and neck pain were not relieved, and the blood culture was negative. Three days after admission, plain computed tomography (CT) of the neck revealed spherical crown-shaped calcifications surrounding the odontoid process (Fig. 1A), and median sagittal sections highlighted linear calcification (Fig. 1B). In addition, slight posterior bulging of the intervertebral discs of C4/5 and C5/6 was observed, likely due to reduced ligamentous support, contributing to segmental instability and disc protrusion (Fig. 1C). Unfortunately, no further MRI of the cervical vertebrae was performed to evaluate the neck due to economic reasons. However, radiography of the knee joints revealed significant calcium deposits around the ligaments (Fig. 1D), suggesting an underlying crystalline deposition. These results align with the CDS diagnosis. After the administration of celecoxib, colchicine, and a short course of corticosteroids for 1 week, the patient’s symptoms were relieved, and no pain was observed during the outpatient follow-up after 2 weeks. ESR and CRP levels returned to within the normal range.

## 3. Discussion

CDS is often regarded as an entity that is not sufficiently acknowledged in clinical practice but should be considered in patients presenting with acute neck or occipital pain, particularly when accompanied by fever, elevated inflammatory markers, and widespread peripheral chondrocalcinosis. By presenting this case, we aimed to raise awareness of CDS, particularly when it coexists with chronic CDH in patients with long-standing neck pain, to aid recognition in primary care settings. On the basis of clinical symptoms, it could be misdiagnosed as an intracranial infection, giant cell arteritis, or polymyalgia rheumatica.^[6]^ A summary of CDS differential diagnoses can be found in Table 1. Patients with CDS often present to general practitioners or general physicians, where the diagnosis may be challenging due to nonspecific symptoms of the condition, potentially leading to unnecessary investigations or empiric antibiotic use. The patient, with a long-standing history of CDH, initially sought treatment for neck pain at an outpatient pain clinic, where conservative therapies were ineffective. Lumbar puncture was not performed because of the absence of meningeal signs.

**Table 1:** The main points and diagnostic indicators for identifying CDS.

‌Differentiating disease	‌Key distinguishing features	‌Specific diagnostic indicators
Meningitis	Absence of meningeal signs in CDS; normal CSF findings	CSF analysis, contrast-enhanced MRI
‌Cervical spondylosis	CDS shows “crown-like” calcifications around odontoid process vs disc herniation	3D-CT demonstrating peridental calcifications
‌Polymyalgia rheumatica (PMR)	CDS pain localized to C1-C2, PMR manifests as symmetric proximal muscle pain	Musculoskeletal ultrasound, Temporal artery biopsy
‌Rheumatoid arthritis (RA)	CDS lacks symmetric joint destruction; RA patients show >70% RF positivity	Anti-CCP testing, Hand X-ray grading
‌Metastatic spinal tumors	CDS calcifications heterogeneous but well-defined; metastases show osteolytic/osteoblastic destruction	PET-CT metabolic activity, Tumor markers
‌Retropharyngeal abscess	No pharyngeal swelling/dysphagia in CDS; abscess often has infectious source	Contrast-enhanced CT showing fluid collection
‌Osteomyelitis	CDS has no bone destruction; osteomyelitis demonstrates sequestrum + periosteal reaction	Blood cultures, MRI bone marrow edema
Ankylosing spondylitis (AS)	CDS lacks sacroiliitis; >90% HLA-B27 positivity in AS	Sacroiliac joint MRI, HLA-B27 testing
‌Giant cell arteritis (GCA)	No temporal artery tenderness in CDS; GCA associated with vision loss/jaw claudication	Temporal artery biopsy, vascular ultrasound

CCP = cyclic citrullinated peptide, CDS = crowned dens syndrome, CSF = cerebrospinal fluid, CT = computed tomography, MRI = magnetic resonance imaging

Cervical CT scans and elevated levels of inflammatory markers are useful for the diagnosis of CDS. Plain CT, which targets the atlantoaxial joint, has become an established diagnostic method for CDS.^[7]^ Furthermore, CT is helpful in differentiating the underlying causes of posterior neck pain, such as tumor metastasis or fracture.^[8]^ In this case, CT of the cervical vertebrae revealed calcium crystal deposition in the ligaments and structures around the odontoid, which caused stiffness and acute neck pain. The CT results simultaneously showed that the patient’s cervical intervertebral disc protrusion was consistent with the previous medical history. Owing to the patient’s economic position, we were unable to complete cervical MRI to further assess cervical intervertebral disc protrusion. The elevated ESR and CRP levels, accompanied by characteristic cervical vertebral changes revealed by the CT scans, we attributed the patient’s acute neck pain to CDS.

In 1985, Bouvet et al first discussed a patient with fever and neck pain resulting from crystalline calcium deposits around the odontoid.^[1]^ CDS is not rare, but can be overlooked. The occurrence of CDS is more prevalent in older women, and it increases with age.^[3]^ Goto et al reported a study of 40 patients diagnosed with CDS and revealed that more than half of the patients were female, and almost two-thirds were aged >70 years.^[9]^ Genetic factors, trauma, inflammation, and the presence of other diseases are also known to affect the progression of CDS.^[10]^ In addition, we collected the clinicopathologic features of in the latest literatures that have been published recently^[2,11–16]^ (Table 2).

**Table 2:** The specific details of the CDS-related cases that were recently published.

Article title	‌Author/Year	‌Patient characteristics	‌Clinical manifestations	‌Imaging features	‌Treatment	‌Outcome and misdiagnosis issues
An uncommon presentation of Crowned dens syndrome without systemic inflammation	Sakhamuru P et al (2025)	74-year-old male	Chronic neck pain, limited mobility; no systemic inflammation	CT: Peridental calcifications, C1-C2 joint erosive changes	NSAIDs + Physical therapy	Significant symptom improvement; highlights CT’s diagnostic value
Cervical epidural abscess mimicking Crowned dens syndrome	Okumura N et al. (2025)	88-year-old female	Acute neck pain, fever; comorbid infective endocarditis	MRI: Peridental abscess (misdiagnosed as CDS)	Antibiotics	Emphasizes CDS vs infectious disease differentials
Clinical features and diagnostic challenges in crowned dens syndrome	Hamdan O et al (2025)	Mean age 73.1, balanced gender	Neck pain (96.8%), fever (80.4%); elevated CRP/ESR (>80%)	CT abnormalities (97.3%, gold standard), MRI sensitivity lower (68.4%)	NSAIDs (62.5%)	96.6% improvement; 21.4% initially misdiagnosed as meningitis
Crowned dens syndrome presenting as failure to thrive in a patient with moderate cognitive impairment	Castellanos G et al (2025)	68-year-old male (cognitive impairment)	Generalized weakness as initial symptom, later acute neck pain	CT: Confirmed peridental calcifications	Corticosteroids	Rapid symptom relief; atypical presentations increase misdiagnosis risk
Crowned dens syndrome triggered by dental treatment	Morita S et al (2025)	Age unspecified	Neck pain and fever post procedure	CT: Typical calcifications	Not specified	Suggests local physical stress may trigger CDS
Case report: Crowned dens syndrome in a patient with seronegative rheumatoid arthritis	Anonymous (2025)	Comorbid seronegative RA	Acute neck pain, fever; overlapping meningitis symptoms	CT: Peridental calcifications	Not specified	Avoid unnecessary lumbar puncture; differentiate from RA flares
Crowned dens syndrome: a rare differential diagnosis of meningitis	Palmowski A et al (2025)	84-year-old female	General weakness, chills, cephalgia, and neck pain	CT: Typical calcifications	Not specified	Initially misdiagnosed as meningitis

CDS = crowned dens syndrome, CT = computed tomography, MRI = magnetic resonance imaging, NSAID = nonsteroidal anti-inflammatory drug, RA = rheumatoid arthritis.

Calcium pyrophosphate dehydrate and hydroxyapatite are the main crystal components that result in CDS.^[15]^ Calcium deposition in the cervical vertebrae has been shown to correlate with peripheral chondrocalcinosis. Calcium deposition around the odontoid process leads to calcification and inflammation of the surrounding ligaments, which may cause posterior neck pain and stiffness during rotation. Evidence of calcium crystalline deposition in other joints, such as the knee, shoulder, elbow, and wrist, supports CDS diagnosis. In this case, radiography of the knee joints revealed calcium deposits in the cartilage and ligaments, with no apparent alteration, deformation, or destruction of the joints. Imaging findings suggest that calcium crystal deposition in the peripheral joints may be present, even in asymptomatic patients, and should be considered during assessment.

The current treatment for CDS remains controversial, and nonsteroidal anti-inflammatory drugs are widely used to alleviate pain and inflammation in patients with acute neck pain. A limited course of corticosteroids has also been prescribed for the acute phase, while colchicine is optimal for some refractory patients.^[8]^ With regard to the age of our patient, celecoxib, colchicine, and a short course of corticosteroids were administered simultaneously. Her temperature quickly decreased, and the neck pain and restricted cervical movement were significantly relieved after 5 days. In addition, the levels of inflammatory markers, including CRP and ESR, decreased rapidly and approached normal levels within 7 days.

In conclusion, CDS should be considered in older patients presenting with fever, posterior neck pain, and limited neck movement, as early cervical CT can aid in diagnosis and help prevent unnecessary procedures or antibiotic use.

## Acknowledgments

We would also like to thank Editage (www.editage.cn) for English language editing.

## Author contributions

**Formal analysis:** Yu Chen.

**Funding acquisition:** Lan Cheng.

**Writing – review & editing:** Lianjie Li.
